# Circulating Carbonic Anhydrase IX and Antiangiogenic Therapy in Breast Cancer

**DOI:** 10.1155/2016/9810383

**Published:** 2016-01-28

**Authors:** Ursa Brown-Glaberman, Marilyn Marron, Pavani Chalasani, Robert Livingston, Maria Iannone, Jennifer Specht, Alison T. Stopeck

**Affiliations:** ^1^University of New Mexico Cancer Center, Albuquerque, NM 87131, USA; ^2^University of Arizona Cancer Center, Tucson, AZ 85719, USA; ^3^Fred Hutchinson University of Washington Cancer Consortium, Seattle, WA 98019, USA; ^4^Stony Brook Cancer Center, SUNY Stony Brook, Stony Brook, NY 11794, USA

## Abstract

*Introduction*. Carbonic anhydrase IX (CAIX) is a hypoxia regulated metalloenzyme integral to maintaining cellular pH. Increased CAIX expression is associated with poor prognosis in breast cancer. To explore CAIX as a biomarker for breast cancer therapies, we measured plasma CAIX levels in healthy control subjects and in breast cancer patients.* Methods*. In control subjects we evaluated plasma CAIX stability via commercially available ELISA. We then similarly quantified plasma CAIX levels in (1) locally advanced breast cancer (LABC) patients treated with neoadjuvant paclitaxel + sunitinib (T + S) followed by doxorubicin and cyclophosphamide (AC); (2) metastatic breast cancer (MBC) patients treated with systemic chemotherapy.* Results*. Plasma CAIX levels were stable at room temperature for at least 48 hours in control subjects. Mean baseline plasma CAIX levels were lower in controls compared to patients with LABC or MBC. In LABC, CAIX levels rose significantly in response to administration of antiangiogenic therapy (T + S) (*p* = 0.02) but not AC (*p* = 0.37). In patients with MBC treated without an antiangiogenic agent CAIX levels did not change with therapy.* Conclusions*. Our results suggest that CAIX may be an easily obtained, stable measure of tumor associated hypoxia as well as a useful pharmacodynamic biomarker for antiangiogenic therapy.

## 1. Introduction

Carbonic anhydrase IX (CAIX), one of the 15 carbonic anhydrase (CA) isoforms found in humans, is a membranous metalloenzyme that facilitates the reversible hydration of carbon dioxide to bicarbonate and protons [1]. CAIX is regulated by hypoxia inducible factor-1 alpha (HIF-1*α*) and is essential for the elimination of acid loads generated by glycolysis. Thus, CAIX plays a major role in maintaining the pH gradient between cells and their extracellular space [2]. An emerging hallmark of cancer is aberrant tumor metabolism, typically characterized by a reliance on glycolysis rather than oxidative phosphorylation [3]. The increased quantities of lactate generated by this aberrant metabolism force tumor cells to acquire adaptive mechanisms to avoid excessive acidosis and perturbations in intracellular pH (pHi). CAs are integral to this process by facilitating proton export and bicarbonate recycling. CAIX is unique among the CA isoforms in that it is seldom expressed in normal tissue and is directly linked to cellular hypoxia, with tumoral expression restricted to perinecrotic or hypoxic regions [1].

Carbonic anhydrase IX, previously referred to as the membrane antigen MN or G250MN, was originally identified in HeLa cells [4]. CAIX mRNA is expressed in >95% of clear cell renal cell carcinomas (ccRCC) [5]. ccRCC is unique among the solid tumors in that HIF-1*α* activity and in turn CAIX expression are no longer tied directly to hypoxia but rather to loss of functional Von Hippel Lindau (VHL) protein. This is thought to explain the near ubiquitous expression of CAIX in ccRCC [6]. Multiple studies have evaluated the prognostic significance of tissue CAIX expression in ccRCC with the majority suggesting that low CAIX expression is associated with worse outcomes [7]. Stewart et al. evaluated CAIX protein expression in ccRCC tumors before and after exposure to vascular endothelial growth factor- (VEGF-) targeted tyrosine kinase inhibitor (TKI) therapy (sunitinib or pazopanib) [8]. They demonstrated that tissue CAIX expression rose following VEGF-targeted therapy and that those patients with high tissue CAIX expression after therapy had improved outcomes, suggesting that CAIX may be a predictive biomarker for antiangiogenic therapy [8].

Carbonic anhydrase IX expression has similarly been confirmed by immunohistochemistry (IHC) in other solid tumor types, including breast cancer [9, 10]. Unlike in ccRCC, high CAIX tumor expression has been associated with poor prognosis and chemoresistance in multiple cancer types, including cervical, lung, head and neck, and breast cancer [10–13].

Recognition that CAIX was shed from propagated cancer cells into culture media led to interest in evaluating CAIX levels in the serum and urine of cancer patients. Závada et al. first described increased expression of the soluble, extracellular CAIX domain in the serum and urine of 50 patients with renal cell carcinoma [14]. In this paper, we describe measurement of circulating plasma CAIX in healthy controls and report its utility as a potential biomarker for antiangiogenic therapy in breast cancer patients.

## 2. Patients and Controls

To determine CAIX stability in plasma, we evaluated CAIX levels in ten healthy control subjects (five female and five male patients, age range 26–67 years) at time 0 and then 6 months later. We then quantified plasma CAIX levels from two breast cancer patient cohorts treated on chemotherapeutic trials. The first cohort consisted of 57 women with HER2 negative locally advanced or inflammatory breast cancer (LABC) treated in the neoadjuvant setting with paclitaxel in combination with the antiangiogenic VEGF-targeted TKI, sunitinib, for 12 weeks followed by continuous anthracycline/cyclophosphamide (AC, weekly doxorubicin and daily oral cyclophosphamide with granulocyte colony-stimulating factor support) for 15 weeks (patient population further described in [15]). Plasma was collected at baseline, after completion of paclitaxel plus sunitinib (T + S) therapy, and at the completion of AC prior to surgery. The second patient cohort consisted of 23 women treated for metastatic breast cancer (MBC) with either irinotecan + etoposide or weekly paclitaxel + a novel immunomodulatory agent. The metastatic patient population was heterogeneous with regard to hormone receptor and HER2 status, as well as number of prior lines of therapy. Plasma samples were collected at baseline and following 4–6 weeks of systemic chemotherapy.

## 3. Methods

For all samples, plasma CAIX was quantified using an enzyme-linked immunosorbent assay (ELISA) kit (Quantikine Human Carbonic Anhydrase IX/CA9 Immunoassay, R&D Systems). Plasma CAIX was quantified in duplicate for control subjects and in triplicate for breast cancer patients. Blood from control subjects was collected with heparin and acid citrate dextrose (ACD) as anticoagulant and analyzed at preplanned time points (within 1 hour, 24 hours, and 48 hours after phlebotomy). For breast cancer patients, plasma was collected in ACD (LABC and subset MBC) or in heparin (subset MCB) and stored at −80°C for batch analysis.

Plasma CAIX mean, median, and range were determined for each group and at each time point. Differences in CAIX levels between baseline and follow-up were compared and statistical significance was determined by paired *t*-test or one-way ANOVA analysis with *p* values ≤0.05 considered significant. In the LABC and MBC groups, patients were divided by the median baseline plasma CAIX level into low and high groups. The median level was chosen as the cut-point as it provides the most power assuming a continuous effect across the spectrum of values, for ease of interpretation and to avoid testing multiple cut-points.

## 4. Results

### 4.1. Healthy Control Subjects

In the healthy control subjects, we first evaluated the effect of heparin versus ACD anticoagulant on plasma CAIX levels. Subsequently, we evaluated the plasma stability of CAIX by quantifying levels within 1 hour as well as at 24 and 48 hours after phlebotomy (Table 1). Intrapatient plasma CAIX levels from blood anticoagulated with ACD were highly correlated with levels from blood anticoagulated with heparin (*r* = 0.98). However, CAIX levels were on average slightly higher (4.6 pg/mL) in blood anticoagulated with heparin compared to ACD (*p* = 0.0003). No significant differences were observed in CAIX levels obtained from plasma collected, spun, and aliquoted within 1 hr after phlebotomy compared to plasma assayed after the blood had remained at room temperature for 24 and 48 hours prior to being spun, aliquoted, and frozen down (heparin *p* = 0.97, ACD *p* = 0.98). Plasma CAIX levels were also remeasured at six months in five of the control subjects (ACD only) with no differences observed in the repeat measurements (*p* = 0.9). While there was a trend toward higher CAIX levels in females when compared to males (mean level 26.2 versus 14.9 pg/mL), this difference was not statistically significant (*p* = 0.1).

### 4.2. Breast Cancer Patients

**Figure 1:**
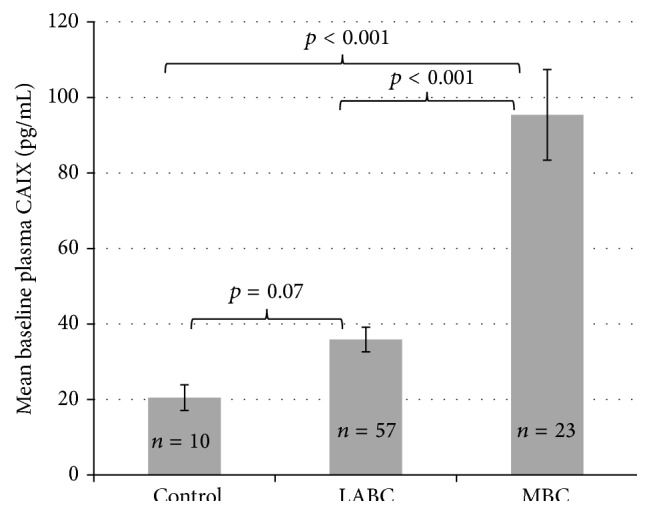
Baseline CAIX levels. Patients with MBC had significantly higher baseline CAIX levels compared to controls and to patients with LABC. LABC: locally advanced breast cancer; MBC: metastatic breast cancer.

In patients with breast cancer, plasma CAIX levels were evaluated at baseline as well as longitudinally in response to the administration of antitumor therapies. In patients with LABC as well as MBC, a wide range of baseline CAIX levels was observed (Table 2). Patients with MBC had significantly higher baseline CAIX levels (mean 90.7 pg/mL) compared to patients with LABC (mean 34.0 pg/mL) or healthy controls (mean 20.5 pg/mL) (Figure 1).

**Figure 2:**
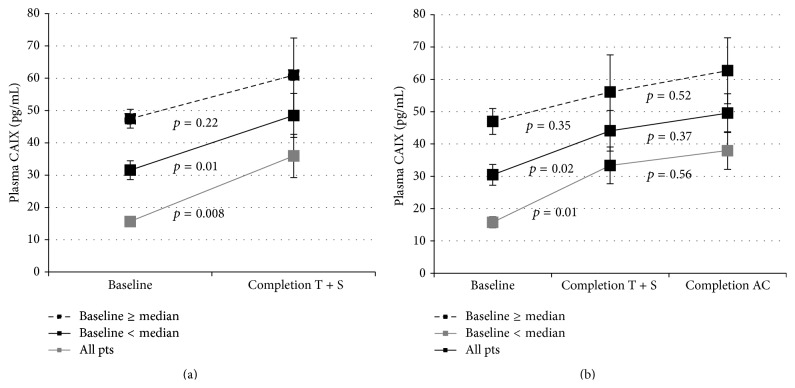
Plasma CAIX levels (pg/mL) in LABC patients in response to treatment. In patients with LABC, CAIX rose significantly in response to T + S (*p* = 0.01) but not further with anthracycline based therapy (*p* = 0.37). The rise in response to T + S was primarily in patients with baseline levels below the median. Paired data, (a) *n* = 46, (b) *n* = 34.

**Figure 3:**
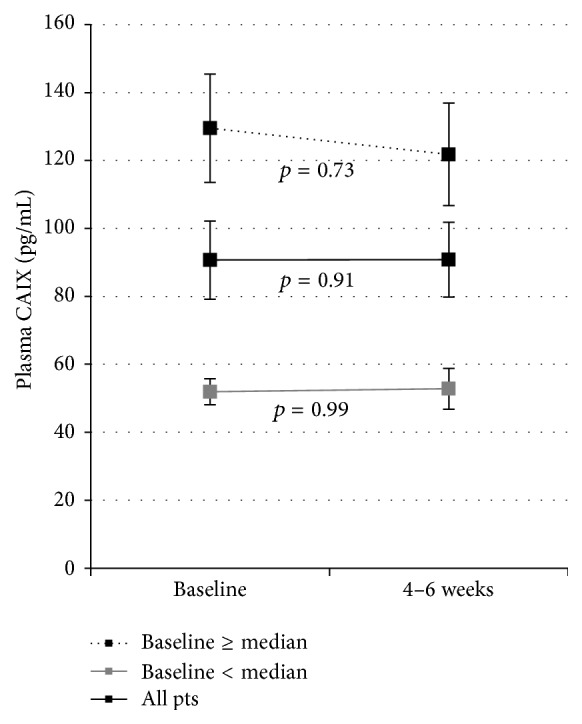
Plasma CAIX levels (pg/mL) in MBC patients in response to treatment. In patients with MBC treated with cytotoxic chemotherapies (without an antiangiogenic agent) CAIX levels did not change in response to therapy. Paired data, *n* = 20.

In patients with LABC, CAIX levels rose significantly in response to the administration of paclitaxel plus sunitinib (*p* = 0.02) but not to anthracycline based therapy (*p* = 0.37) (Figure 2(b)). The rise in CAIX in response to paclitaxel plus sunitinib was statistically significant only in patients with lower baseline CAIX levels (*p* = 0.008) (Figure 2(a)). In patients with MBC treated with cytotoxic chemotherapies (without an antiangiogenic agent), CAIX levels did not change in response to therapy administration (Figure 3).

In patients with LABC, elevated baseline plasma CAIX levels were also associated with triple negative histology and a decreased clinical/pathologic response to neoadjuvant chemotherapy. Patients with a baseline CAIX level below the median had a complete pathologic response (pCR) rate of 44.8% at the time of surgery compared to a pCR rate of 11.5% in those with a baseline CAIX level greater or equal to the median value (*p* = 0.008) (manuscript in preparation; see [16]).

## 5. Discussion

Biomarkers for response to antiangiogenic therapy remain elusive despite the extensive use of antiangiogenic therapies in cancer patients and the intense search for predictive markers. Circulating plasma CAIX has several attributes suggesting it may be a promising biomarker for antiangiogenic therapies in breast cancer. First, it is exclusively regulated by HIF-1 alpha and thus increases rapidly in response to tissue sensed hypoxia. CAIX is also crucial for maintaining the cellular pH in tumor cells that have switched to a glycolytic phenotype and thus a marker for tumor cells that have already adapted to a hypoxic, acidotic environment.

In this paper, we extended the findings of Wind et al. who previously evaluated two commercially available ELISA kits for measuring circulating CAIX and found that the R&D Systems kit was suitable for use with both serum and plasma collected from blood anticoagulated with EDTA with reliable, reproducible results [17]. We have also shown that CAIX levels are stable for at least 48 hours after phlebotomy, assessable in plasma anticoagulated with either ACD or heparin, and remarkably stable over time.

Elevated tissue and circulating CAIX levels have been associated with a more aggressive phenotype, resistance to chemotherapy, and poor prognosis in breast cancer. Chia et al. initially characterized CAIX expression in primary breast tumors from 103 women [10]. CAIX expression was detectable in 48% of cases as strong membranous staining within epithelial tumor cells. The pattern of expression was typically limited to tumor cells immediately adjacent to areas of necrosis [10]. CAIX expression was associated with the presence of tumor necrosis, higher grade, loss of estrogen receptor (ER) expression, and decreased recurrence-free and overall survival [10]. The correlation between elevated CAIX expression, aggressive histology, and poor prognosis has subsequently been confirmed in several series with the highest expression observed in basal-type breast cancers (51%) and the lowest in luminal A subtypes (8%) [18–21]. High tumor CAIX expression may also correlate with resistance to chemotherapy as patients with early stage basal-like breast cancer positive for CAIX expression had significantly shorter survival when compared to patients with CAIX-negative basal-like breast cancer (*p* = 0.03) [22]. Similarly, breast tumors that expressed higher levels of CAIX had lower pathologic complete response (pCR) rates when treated with neoadjuvant anthracycline/taxane based therapy [23].

There are multiple hypotheses that may explain the association between high CAIX expression and poor outcome in breast cancer. CAIX expression is closely linked to tissue hypoxia and acidosis suggesting that its upregulation is part of the tumor's adaptation to survive and even thrive under hypoxic conditions, including those generated by antiangiogenic therapies [19]. Tumors that develop a glycolytic-acid resistant phenotype require upregulation of CAIX to maintain their intracellular pH while fostering acidification of the extracellular space. This acidity contributes to breakdown of the extracellular matrix increasing the tumor's invasive potential [19, 24]. Increased intratumoral hypoxia may also contribute to genomic instability and the loss of genomic integrity [25] as elevated CAIX expression has been associated with loss of BRCA1 function [26, 27]. CAIX has also been proposed to influence the expansion and survival of breast cancer stem cells under hypoxic conditions [28].

The clinical significance of circulating CAIX levels in patients with metastatic and early stage breast cancer has been previously reported [24, 29]. Müller et al. prospectively examined serum CAIX levels from a heterogeneous group of 253 patients with metastatic breast cancer [24]. Patients with elevated CAIX levels (35% of patients) had a significantly worse median progression-free survival (PFS) and overall survival (OS) (*p* < 0.01). On multivariate analysis with established prognostic factors, the presence of circulating tumor cells, the line of therapy, and elevated serum CAIX remained independent predictors of OS [24].

Schütze et al. similarly evaluated serum CAIX and tumor CAIX expression by microarray analysis in patients with stage I–III breast cancer [29]. In the 76 patients with both tissue CAIX RNA and serum CAIX levels, there was no correlation between serum levels and tumor RNA expression (*p* = 0.332) or between CAIX and overall survival. The authors suggested this seemingly contradictory result may be related to differences in treatment after recurrence, the relatively small size of the patient cohort, and the different techniques used for tissue CAIX analyses (tissue RNA as opposed to IHC). However, neither study examined changes in circulating CAIX in response to chemotherapy.

Our work in breast cancer patients further expands on these results and provides additional patient data showing that plasma CAIX levels are elevated in patients with MBC when compared to those with LABC or to healthy controls and that elevated CAIX levels may predict tumors adapted to a glycolytic, acidic, and hypoxic environment. In addition, our results show that changes in circulating CAIX levels are observed in response to the administration of antiangiogenic but not cytotoxic chemotherapy. Further work is needed to explore the relationship between circulating CAIX levels and clinical outcome in patients with breast cancer.

The wide range in baseline CAIX levels in patients with MBC and LABC may in part explain the varied response historically observed with therapies targeting angiogenesis. Those tumors adapted to a hypoxic/acidotic environment, as suggested by elevated baseline CAIX levels, may be less sensitive to such therapies as they have developed compensatory mechanisms to survive in devascularized conditions. Supporting this hypothesis, Shan et al. demonstrated that elevated tissue CAIX expression in primary breast cancer is associated with lower tumor microvessel density and increased tumor necrosis [30]. Additionally, knock-down of CAIX expression in colon cancer cell lines was found to enhance the effects of bevacizumab treatment, perhaps by preventing tumors from adapting to the increased hypoxia induced by anti-VEGF treatment [31].

Multiple selective CAIX inhibitors are currently under evaluation in the preclinical setting [32, 33]. SLC-0111 is an orally bioavailable, highly selective small molecule inhibitor of CAIX and CAXII. Daily oral administration of SLC-0111 to mice harboring MDA-MB-231 LM2-4 orthotopic human breast tumors (known to be ER/PR and HER2 negative, CAIX-positive) resulted in significant, dose-dependent reductions in tumor growth [34]. Furthermore, treatment with SLC-0111 in combination with paclitaxel was significantly more effective at inhibiting tumor growth compared to either treatment alone, with no additional toxicity. Phase I study is currently underway investigating the safety, tolerability, and pharmacokinetics of SLC-0111 in patients with advanced solid tumors (clinicaltrials.gov, NCT02215850).

In conclusion, our results validate circulating CAIX as a robust and easily measurable biomarker of hypoxia and HIF-1*α* upregulation that may prove useful in identifying patients unlikely to benefit from antiangiogenic therapy. Similarly, CAIX may be a future target of interest in breast cancer with the potential of augmenting the effectiveness of antiangiogenic therapy by preventing tumors from surviving in and adapting to hypoxia.

## Figures and Tables

**Table 1:** CAIX levels in healthy control subjects (*n* = 10).

Timing plasma collection^*∗*^	Anticoagulant^#^	CAIX, pg/mL		
		Mean	Median	Range
Baseline, within 1 hr after phlebotomy	ACD	20.5	17.4	8.8–41.2
Baseline, 24 hrs after phlebotomy	ACD	21.1	16.4	8.7–36.4
Baseline, 48 hrs after phlebotomy	ACD	20.3	16.7	8.6–36.8
6-month follow-up, within 1 hr after phlebotomy	ACD	22.48	26.9	9.5–28.0
Baseline, within 1 hr after phlebotomy	Heparin	25.1	21.2	11.9–45.9
Baseline, 24 hrs after phlebotomy	Heparin	24.9	21.2	11.1–46.1
Baseline, 48 hrs after phlebotomy	Heparin	26.2	22.7	10.8–50.2

^*∗*^Referring to the time blood remained at room temperature prior to plasma separation, aliquoting, and freezing. ^#^CAIX levels were on average 4.6 pg/mL higher in blood anticoagulated with heparin compared to ACD (*p* = 0.0003). ACD: acid citrate dextrose; hr: hour; hrs: hours.

**Table 2:** Plasma CAIX in breast cancer patients.

Patient population	Chemotherapy	Time points	CAIX, pg/mL		
			Mean	Median	Range
LABC	Neoadjuvant paclitaxel (T) + sunitinib (S) × 12 weeks followed by anthracycline (AC) × 15 weeks	Baseline (*n* = 57)	34.0	26.0	0–155
		Following T + S (*n* = 46)	48.4	28.5	0–239
		Following AC (*n* = 34)	49.6	42.2	15–172

MBC	Irinotecan + etoposide or paclitaxel + immunomodulatory agent	Baseline (*n* = 23)	90.7	76.9	31.2–254.7
		Following 4–6 weeks of	90.7	74.3	27.2–224
		chemotherapy (*n* = 20)			

LABC: locally advanced breast cancer; MBC: metastatic breast cancer.

## Abbreviations

AC: Doxorubicin plus cyclophosphamide
ACD: Acid citrate dextrose
CA: Carbonic anhydrase
CAIX: Carbonic anhydrase IX
CAXII: Carbonic anhydrase XII
ccRCC: Clear cell renal cell carcinoma
ELISA: Enzyme-linked immunosorbent assay
ER: Estrogen receptor
HIF-1*α*: Hypoxia inducible factor-1 alpha
IHC: Immunohistochemistry
LABC: Locally advanced breast cancer
MBC: Metastatic breast cancer
OS: Overall survival
pCR: Pathologic complete response
PFS: Progression-free survival
pHi: Intracellular pH
PR: Progesterone receptor
T + S: Paclitaxel plus sunitinib
TKI: Tyrosine kinase inhibitor
VEGF: Vascular endothelial growth factor
VHL: Von Hippel Lindau.
